# Alternative paths in HIV-1 targeted human signal transduction pathways

**DOI:** 10.1186/1471-2164-10-S3-S30

**Published:** 2009-12-03

**Authors:** Sivaraman Balakrishnan, Oznur Tastan, Jaime Carbonell, Judith Klein-Seetharaman

**Affiliations:** 1 Language Technologies Institute, School of Computer Science, Carnegie Mellon University, 5000 Forbes Avenue, Pittsburgh, PA15213, USA; 2 Department of Structural Biology, University of Pittsburgh School of Medicine, 3501 Fifth Avenue, Pittsburgh, PA15260, USA

## Abstract

**Background:**

Human immunodeficiency virus-1 (HIV-1) has a minimal genome of only 9 genes, which encode 15 proteins. HIV-1 thus depends on the human host for virtually every aspect of its life cycle. The universal language of communication in biological systems, including between pathogen and host, is via signal transduction pathways. The fundamental units of these pathways are protein protein interactions. Understanding the functional significance of HIV-1, human interactions requires viewing them in the context of human signal transduction pathways.

**Results:**

Integration of HIV-1, human interactions with known signal transduction pathways indicates that the majority of known human pathways have the potential to be effected through at least one interaction with an HIV-1 protein at some point during the HIV-1 life cycle. For each pathway, we define simple paths between start points (i.e. no edges going into a node) and end points (i.e. no edges leaving a node). We then identify the paths that pass through human proteins that interact with HIV-1 proteins. We supplement the combined map with functional information, including which proteins are known drug targets and which proteins contribute significantly to HIV-1 function as revealed by recent siRNA screens. We find that there are often alternative paths starting and ending at the same proteins but circumventing the intermediate steps disrupted by HIV-1.

**Conclusion:**

A mapping of HIV-1, human interactions to human signal transduction pathways is presented here to link interactions with functions. We proposed a new way of analyzing the virus host interactions by identifying HIV-1 targets as well as alternative paths bypassing the HIV-1 targeted steps. This approach yields numerous experimentally testable hypotheses on how HIV-1 function may be compromised and human cellular function restored by pharmacological approaches. We are making the full set of pathway analysis results available to the community.

## Background

Human immunodeficiency virus-1 (HIV-1) is the causative agent for acquired immune deficiency syndrome (AIDS), a world-wide epidemic that according to statistics from the world health organization has resulted in more than 2 million deaths; 33 million people are living with HIV, including 2 million at the age of 15 years and under. While current antiviral medication has dramatically improved the life expectancy of AIDS patients, the medicines are not available to everyone and drug resistance and side effects are increasingly recognized problems. Thus, novel avenues to anti-HIV-1 drug discovery are needed. The availability of large-scale transcriptomic [1], proteomic [2-4] and phenotypic [5-7] information for HIV-1 provide an opportunity to explore drug discovery through systems biology. Despite the small genome of only 9 genes, which encode a slightly larger number of proteins (15) due to post-translational cleavage, more than 2500 interactions between human and HIV-1 proteins have been reported in the literature [2-4,8]. This data has been recently used to create a human, HIV-1 interactome map via information integration of numerous features such as gene expression, domain and motif identification, tissue distribution, functional annotation, subcellular localization and human network features and HIV-1's mimicry of human protein binding partners [9].

The general function of these interactions is for the virus to subvert the human cellular machinery for its purposes. Multiple mechanisms are employed by the virus, including manipulation of endo- and exocytosis, nuclear transport, transcription and translation, protein degradation and autophagocytosis. In all cases the virus must communicate with the host cell, and this communication typically takes place through interactions between HIV-1 and human host proteins. Communication through protein interaction is the universal language of cells; chains of interactions referred to as signal transduction pathways allow cells to respond to the environment. The virus simply needs to intercept these chains at one or more positions to "talk" to the cell. To systematically generate viable hypotheses on causal relationships between the phenotypic information of which proteins are critical for virus function and the protein interactions, a coherent picture of cellular pathways has to be developed in which the position of the proteins important for HIV-1 function are highlighted. Within this global picture, it should be possible to identify the points of interception by the virus through specific physical interactions with human proteins. Towards this goal, we recently presented an integration of the literature-reported [3] and predicted [9] interaction maps with known signal transduction pathway maps [10].

**Figure 1:**
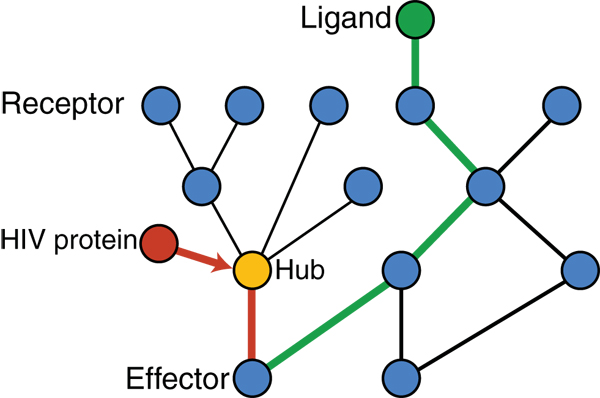
**Schematic of the approach for analysis of HIV-1 targeted human signal transduction pathways**. HIV-1 interception of paths within cellular communication pathways and alternative paths not involving any HIV-1 interactions leading to the same endpoints as the HIV-1 intercepted paths are shown.

In this paper, we propose a novel approach to analyze the HIV-1 intercepted pathways based on identification of paths of connected interactions with directionality within the pathways. The conceptual basis for the approach is as follows. The major features of cellular communication networks are their efficiency and robustness brought about by the use of hubs and redundancy, respectively. In contrast, the virus has to be a minimalist in order to survive and will therefore target the hub proteins, and this is what we (data not shown) and others [11] have observed. Here, we propose the idea that the redundancy may allow circumventing the points of interception by HIV-1 (Figure 1). To lay the groundwork for testing this hypothesis, for each pathway, we define simple paths that start with a protein that does not receive an input from another protein and end with a protein that does not induce a change in any other protein. Through this definition, the approach takes the directionality of interactions in pathways into account. We then identify which of these paths contain proteins that interact with HIV-1 proteins, and find alternative paths between the same start and end points that do not traverse any protein that can interact with an HIV-1 protein. We supplement the combined map with functional information, namely which proteins are known drug targets and which proteins have shown an effect on HIV-1 infectivity and other functions upon siRNA silencing. The putative drug targets in the alternative paths may be up-stream or down-stream of the HIV-1 target, as we include all paths leading from any valid input to any valid output node preserving the directionality of the interactions. Using this approach, we can generate experimentally testable hypotheses on how HIV-1 survival may be compromised by pharmacological approaches providing a new resource to the community that can be used to stimulate mechanistic studies on human-pathogen interactions and HIV-1 drug discovery.

## Results and discussion

### Many human signal transduction pathways may be targeted by HIV-1

To identify HIV-1 targeted and alternative paths, we first determined the intersection between the HIV-1, human interactome and the gathered signal transduction pathways [10]. This requires defining the interactome. We used the following 5 definitions, all directly or indirectly derived from the NIAID database [3]: Group 1 are the most likely direct, physical known interactions based on reported literature [3], while Group 2 refer to more likely indirect interactions reported in the literature [3]. Group 1 and Group 2 interactions are mutually exclusive. (For more details on definition of Group 1 and Group 2, see [9]). In an effort to define a more reliable set of known interactions, we asked expert virologists to annotate the interactions from the literature, primarily focusing on Group 1 interactions. We found that many HIV-1 virologists do not trust the majority of the interactions reported in the literature. Expert validated targets 1 refer to those interactions where at least one expert annotated the interaction as real. Expert validated targets 2 refer to those interactions where all experts agree on their label. Finally, we included all of our predicted interactions[9]. These definitions vary in the degree of confidence that an interaction in this category is real. While the Expert validated targets 2 include the most confident interactions, this dataset is also the smallest, leaving many false negatives. On the other hand, Group 2 and the predicted interactions contain many false positives. Thus, we have to take into account that errors originating from uncertainty in the experimental data will affect our analysis. Note that we do not distinguish between interactions that are obtained from high confidence experimental data such as co-complex crystal structures as compared to more error-prone experiments such as using in vitro pull-down assays.

**Figure 2:**
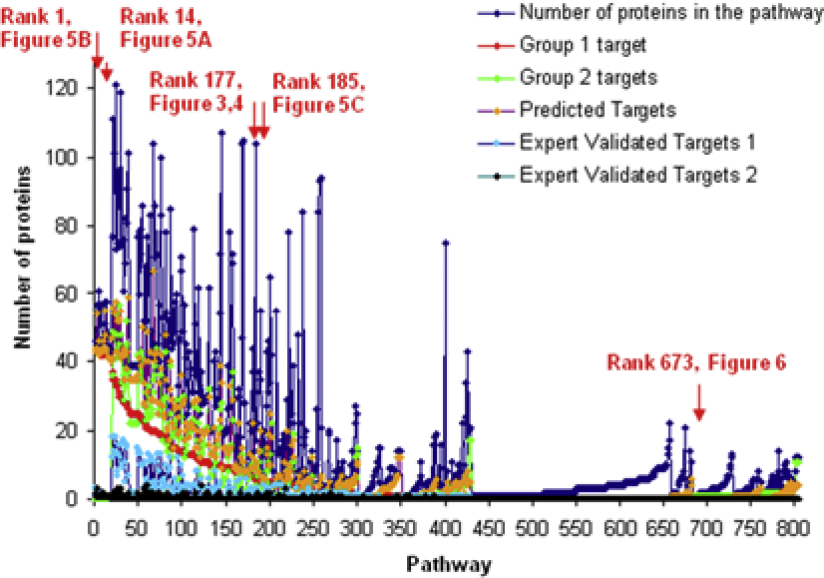
**Number of proteins targeted in signal transduction pathways by HIV-1**. This graph represents the mapping of the NCI PID [12] and Reactome [14] databases with respect to HIV-1 targets obtained from the NIAID database [2] and predicted through information integration [8]. The pathways are sorted by the number of proteins known to interact with HIV-1 per pathway, referred to as Group 1 targets (for details, see Methods). The data is compared to other definitions of HIV-1, human protein pairs: Group 2 targets (more likely functional interactions from the NIAID database), predicted targets [8], Expert validated targets 1 (where any expert annotated group 1 targets), Expert validated targets 2 (where all of the experts annotated Group 1 targets with the same label, i.e. they all agreed that the interaction is real). We also plot the total number of proteins in the pathway for comparison. Highlighted are the pathways that are discussed in this paper, with reference to their rank according to the number of Group 1 targets, and the Figure in which they are illustrated.

An overview of the result of integrating these five interaction networks with known signal transduction pathways (see Methods) are shown for 453 pathways analyzed in Figure 2. The graph shows the number of interactions as a function of pathway, sorted by Group 1 interactions. The majority of known human pathways can potentially be targeted through at least one Group 1 HIV-1, human protein interaction: 277 of 453 pathways analyzed include at least one host factor that interacts with one of the HIV-1 proteins, a number that increases to 303 if predicted host factors are included. Some pathways involve many interactions, but these are not necessarily the largest pathways. Furthermore, some pathways, even the very small ones, include expert validated targets 2, indicating a very high confidence in the interaction and its likely functional relevance.

To obtain an indication as to whether these interactions are possible based on actual translation of the gene transcripts, we investigated their presence in HIV-1 susceptible tissues (see Methods). The number of unique proteins in all pathways combined is 3039. This corresponds to 2956 genes, indicating that in the vast majority of genes, it is only one isoform that has been studied with respect to its interaction with HIV-1 proteins. This set of proteins gives rise to 15161 unique HIV - UniProt pairs, out of which 689 are observed in pathways. 396 of these pairs are targeted by HIV-1 according to group 1. Most of these interactions are possible based on the expression of the human proteins in HIV-1 susceptible tissues: of the 3039 proteins in the pathways, only 85 are expressed in tissues that are not susceptible to HIV-1, while 2728 are in tissues that are susceptible to HIV-1 (for 175 there was no information available and for 51 a corresponding gene could not be identified). The information whether a gene is expressed in a HIV-1 susceptible tissue is provided in Additional File 1 for all pairwise interactions.

### Human network redundancy provides numerous alternative paths to the HIV-1 targeted paths

**Figure 3:**
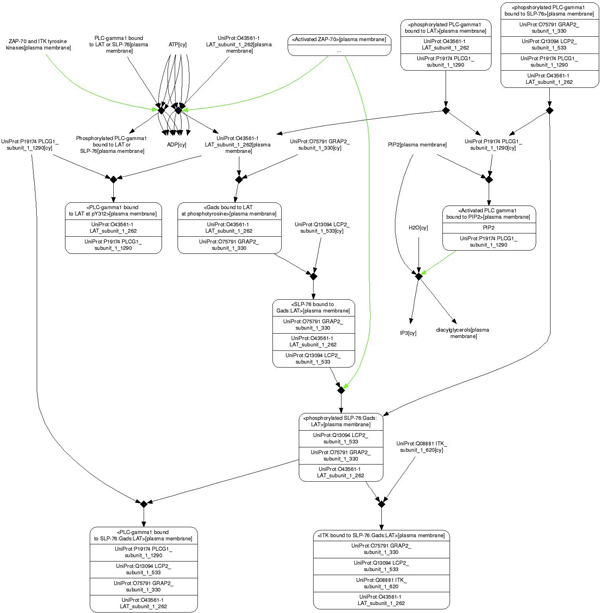
**Generation of second messenger pathway**. This image shows the pathway as it is illustrated in the Reactome Database [15].

**Figure 4:**
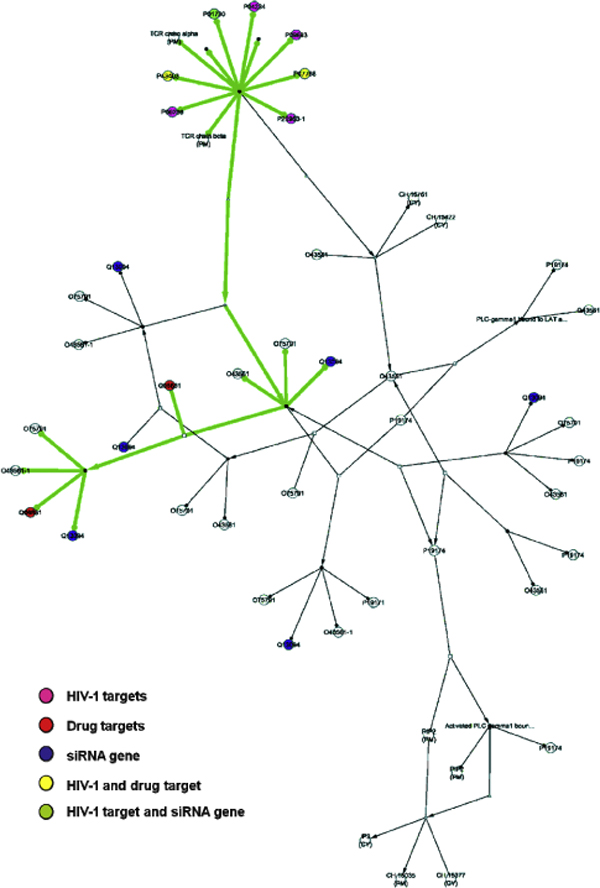
**Generation of second messenger pathway**. This is the same pathway as the one shown in Figure 3, but processed and visualized using cytoscape software [17]. The network of interactions is shown and the proteins that are either targeted by HIV-1, are an siRNA gene or are a drug target are highlighted in color: Purple - HIV-1 target according to Group 1; Blue - siRNA gene; Red - drug target; Yellow - drug, si-RNA and HIV-1 target; Green - si-RNA and drug target; Silver - drug and HIV-1 target; Brown - siRNA and HIV-1 target. Highlighted in green is one path leading from an input to an output node, and which is intercepted by an HIV-1 interaction.

To analyze the integrated pathway maps, for each of the 453 pathways, we define simple paths that start with a protein that does not receive an input from another protein and end with a protein that does not induce a change in any other protein. Searching for simple paths was carried out by a breadth first search algorithm (see Methods). Within these paths, we identify which contain proteins that interact with HIV-1 proteins, and then find alternative paths between the same start and end points that do not traverse any protein that can interact with an HIV-1 protein. We supplement the combined map with functional information, namely which proteins are known drug targets [15] and which proteins have shown an effect on HIV-1 infectivity and other functions upon siRNA silencing [3-5]. This path search yields many potentially interesting results. Table 1 lists example pathways, along with relevant overall path and target statistics. The pathways shown are top-ranked when sorting paths by the maximum number of non-targeted paths that also contain siRNA genes. These are particularly interesting pathways, because if a path that does not contain a direct HIV-1 interaction partner is an alternative path, the presence of an siRNA gene demonstrates that it is already functionally linked to HIV-1 biology, despite the absence of a direct HIV-1 partner. One of these pathways, the generation of second messengers, is illustrated in more detail in Figures 3 and 4. Figure 3 shows the pathway the way it is visualized in the database from which it was downloaded [12]. Figure 4 shows a network representation of the same pathway implemented using cytoscape software [13], highlighting one of the HIV-1 targeted path satisfying our definition (see above) and the positions of HIV-1, siRNA and drug targets in the network. Table 2 lists the proteins in this pathway.

**Table 1:** Overall path statistics on pathways. The table shows the top-ranked pathways from the NCI PID database [12] when sorting paths by the maximum number of non-targeted paths that also contain siRNA genes after applying a filter to only include pathways with alternative paths that contain at least one HIV-1 target, at least one drug target and at least one si-RNA target.

Pathway Name	Number of end points	Number of paths	Non-targeted	Number of proteins	SiRNA	Group 1	Group 2	Predicted	Expert validated targets 1	Expert validated targets 2	Non-targeted + SiRNA genes	Number of drug targets
Activated AMPK stimulates fatty acid oxidation in muscle	6	17	16	5	1	1	1	1	0	0	4	2
^1^Generation of second Messenger molecules	8	34	30	12	2	7	7	9	2	1	2	3
Phosphorylation of Emi1	3	10	8	4	1	1	2	2	0	0	2	1
Activation of BAD and translocation to mitochondria	2	8	3	7	1	1	7	4	0	0	1	2
Mitotic Prometaphase	2	4	2	78	12	4	5	10	1	0	1	1
Polo like kinase mediated events	3	6	2	4	1	2	1	3	2	0	1	1
Notch HLH transcription	2	11	5	3	1	2	1	3	0	0	1	1
Global Genomic NER GG NER	10	13	9	20	5	10	7	13	1	0	1	1
Intrinsic Pathway	2	4	4	17	2	4	2	5	0	0	0	10
Orc1 removal from Chromatin	3	11	1	57	13	43	0	54	1	1	0	8
...												
^2^Cholesterol biosynthesis	7	140	140	8	1	0	0	1	0	0	7	2
^3^EGFR downregulation	12	88	5	17	4	6	4	11	3	1	0	4

^1^Ranked 177^th ^in Figure 2 and shown in Figures 3 and 4.

^2^Ranked 673^rd ^in Figure 2 and shown in Figure 6.

^3^Ranked 185^th ^in Figure 2 and shown in Figure 5C.

**Table 2:** Proteins in generation of the second messenger molecules pathway. This table lists the proteins that are the constituents of the generation of the second messengers pathway (Figure 3 and 4). Column 1 gives the uniprot identifier, column 2 the short gene description, column 3 the gene symbol and column 4 highlights the features of this protein, including whether or not it is a HIV-1, siRNA or drug target.

Uniprot id	Gene description	Gene Symbol	Properties
P07766	CD3e molecule epsilon (CD3-TCR complex)	CD3E	HIV-1 target according to Group 1, Group 2 and predictions, known drug target
Q13094	lymphocyte cytosolic protein 2 (SH2 domain containing leukocyte protein of 76kDa)	LCP2	HIV-1 target according to predictions, siRNA protein
P04234	CD3d molecule delta (CD3-TCR complex)	CD3D	HIV-1 target according to Group 1, Group 2 and predictions
P19174	phospholipase C gamma 1	PLCG1	HIV-1 target according to Group 2, and predictions
O43561	linker for activation of T cells	LAT	None
O75791	GRB2-related adaptor protein 2	GRAP2	None
P01730	CD4 molecule	CD4	HIV-1 target according to Group 1, Group 2, predictions, and experts, siRNA protein
P09693	CD3g molecule gamma (CD3-TCR complex)	CD3G	HIV-1 target according to Group 1, Group 2, and predictions
P43403	zeta-chain (TCR) associated protein kinase 70kDa	ZAP70	HIV-1 target according to Group 1, Group 2, and predictions, known drug target
Q08881	IL2-inducible T-cell kinase	ITK	Known drug target
P06239	lymphocyte-specific protein tyrosine kinase	LCK	HIV-1 target according to Group 1, Group 2, and predictions
P20963	CD247 molecule	CD247	HIV-1 target according to Group 1, predictions and experts

### Degradation and down-regulation pathways are highly targeted

**Figure 5:**
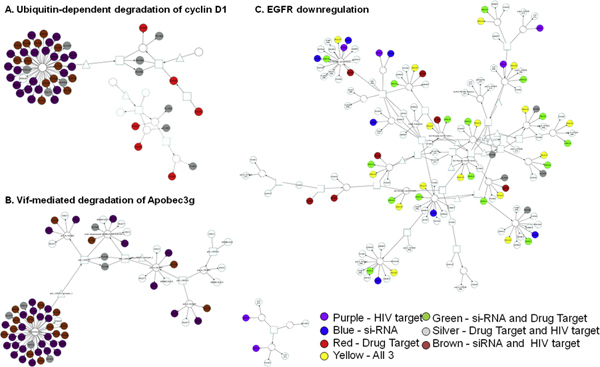
**Degradation and down-regulation pathways containing alternative paths to HIV-1 targeted paths**. A. Ubiquitin dependent degradation of cyclin D1. The colored circle represents the proteasome complex. B. Vif-mediated degradation of Apobec3g. The same proteasome complex shown in A is part of this pathway also. This is an HIV-1 specific pathway as per its listing in the pathway database, and thus already contains HIV-1 proteins as part of the pathway. C. EGFR down-regulation. Here, down-regulation is achieved via internalization. The color coding is as follows: Purple - HIV target (according to G1, except in the cholesterol one where the purple is according to predictions). Blue - si-RNA, Red - Drug Target, Yellow - All 3, Green - si-RNA and Drug, Target, Silver - Drug Target and Group 1, Brown - siRNA and Group 1.

Two of the top-most frequently HIV-1 targeted (according to Group 1 definition) pathways are shown in Figure 5A and 5B. These represent ranks 1 and 14 within the initial plateau in Figure 2. Inspection of the lists of proteins in these pathways (see Additional File 2) shows that the reason for the over-proportionally large fraction of HIV-1 targets in these pathways is the proteasome and ubiquitin targets. These can be observed in many pathways, especially the top ranked ones in Figure 2. In graphs Figure 5A and 5B, the proteasome (the most colored, large complex) is the dominant feature. The graphs look very similar, although one pathway was not specific for HIV-1 (ubiquitin-dependent degradation of cyclin D1), while the other was (Vif-mediated degradation of Apobec3g), demonstrating that our analysis may reveal many more pathways targeted by HIV-1 than those specifically already studied in the context of HIV-1 infection. Even when moving down the ranks, other pathways are also related to degradation and down-regulation. One example is the EGFR down-regulation, shown in Figure 5C. Proteins in this pathway are listed in Table 3. In this pathway, down-regulation is achieved via internalization of the receptor from the surface. This analysis suggests removal of "unwanted" human cellular factors as a major contributor to the disruption of human signaling pathways by HIV-1.

**Table 3:** Proteins in EGFR downregulation pathway. This table lists the proteins that are the constituents of the EGFR downregulation pathway (Figure 5D). Column 1 gives the uniprot identifier, column 2 the short gene description, column 3 the gene symbol and column 4 highlights the features of this protein, including whether or not it is a HIV-1, siRNA or drug target.

Uniprot id	Gene description	Gene Symbol	Properties
O94973	adaptor-related protein complex 2 alpha 2 subunit	AP2A2	HIV-1 target according to Group1, predictions and the experts
Q96B97	SH3-domain kinase binding protein 1	SH3KBP1	HIV-1 target according to predictions
P09496	clathrin light chain (Lca)	CLTA	siRNA protein
P22681	Cas-Br-M (murine) ecotropic retroviral transforming sequence	CBL	HIV-1 target according to Group 2 and predictions
Q9UBC2	epidermal growth factor receptor pathway substrate 15-like 1	EPS15L1	None
P01133	epidermal growth factor (beta-urogastrone)	EGF	HIV-1 target according to Group 1 and predictions, siRNA protein and known drug target
P62993	growth factor receptor-bound protein 2	GRB2	HIV-1 target according to Group 2 and predictions, known drug target
Q00610	clathrin heavy chain (Hc)	CLTC	HIV-1 target according to Group 1 and predictions
Q9Y6I3	epsin 1	EPN1	None
O95782	adaptor-related protein complex 2 alpha 1 subunit	AP2A1	HIV-1 target according to Group 1, predictions and experts
Q99962	SH3-domain GRB2-like 2	SH3GL2	None
P42566	epidermal growth factor receptor pathway substrate 15	EPS15	HIV-1 target according to predictions
P00533	epidermal growth factor receptor (erythroblastic leukemia viral (v-erb-b) oncogene homolog avian)	EGFR	HIV-1 target according to Group 2, and predictions, siRNA protein and known drug target
O14964	hepatocyte growth factor-regulated tyrosine kinase substrate	HGS	HIV-1 target according to Group 2, siRNA protein
P60953	cell division cycle 42 (GTP binding protein 25 kDa)	CDC42	HIV-1 target according to Group 1 and predictions
P62988	ribosomal protein S27a	RPS27A	HIV-1 target according to Group 1, predications and experts, known drug target
O43597	sprouty homolog 2 (Drosophila)	SPRY2	None

### Inhibiting cholesterol biosynthesis as a putative mechanism to inhibit HIV-1

**Figure 6:**
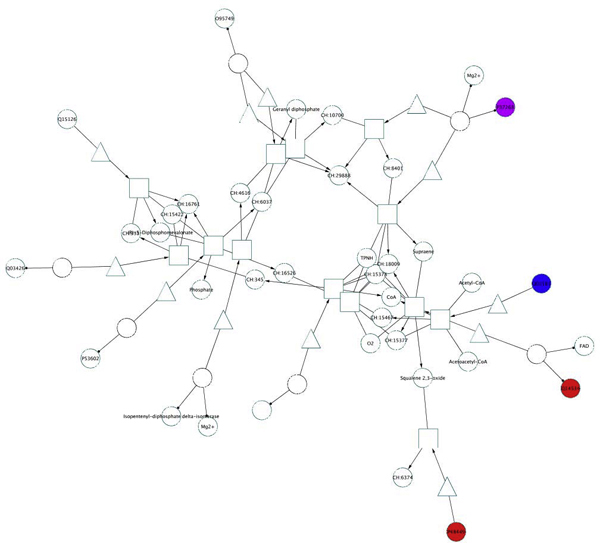
**Cholesterol biosynthesis pathway**. The color coding is as in Figure 5.

The pathways described above are those targeted most extensively according to the Group 1 definition. To demonstrate the utility of also including predictions of novel HIV-1, human protein interactions, we selected the cholesterol biosynthesis pathway, shown in Figure 6. The proteins in this pathway are listed in Table 4. This pathway is not targeted by any of the known interactions, but according to our predictions [9], an HIV-1 protein (Tat) interacts with farnesyl-diphosphate farnesyltransferase 1 (uniprot id P37268/gene symbol FDFT1). The pathway contains two drug targets in alternative paths, lanosterol synthase (2,3-oxidosqualene-lanosterol cyclase, P48449/LSS) and squalene epoxidase (Q14534/SQLE). It also contains a siRNA gene, soluble 3-hydroxy-3-methylglutaryl-Coenzyme A synthase 1 (Q01581/HMGCS1). Thus, the cholesterol synthesis pathway would be a good candidate for drug design because it contains alternative paths with known drug targets and has already been functionally linked to HIV-1 biology through the presence of the siRNA gene. Additional evidence supporting such a functional link is given by numerous statistics showing AIDS patients' increased risk for arteriosclerosis. Furthermore, it was shown experimentally, that the HIV-1 protein Nef blocks the cholesterol efflux pump ABCA1, resulting in cholesterol oil droplets inside cells [14]. Based on these findings, it was previously proposed that activating cholesterol efflux might counteract HIV-1. In the Reactome and NCI pathway databases, ABCA1 appears in two pathways, (1) the RXR and RAR heterodimerization with other nuclear receptor pathway (from NCI), which is part of signaling by the Retinoic Acid receptors pathway and (2) HDL mediated lipid transport (from Reactome). Both pathways are targeted by HIV-1, contain siRNA genes as well as numerous drug targets (14 and 6, respectively) [for details see Supplementary Table S2]. Thus, our analysis provides additional means to explore known HIV-1 interactions in the context of different signaling pathways.

**Table 4:** Cholesterol biosynthesis pathway. This table lists the proteins that are the constituents of the cholesterol biosynthesis pathway (Figure 5E). Column 1 gives the uniprot identifier, column 2 the short gene description, column 3 the gene symbol and column 4 highlights the features of this protein, including whether or not it is a HIV-1, siRNA or drug target.

Uniprot id	Gene description	Gene Symbol	Properties
Q01581	3-hydroxy-3-methylglutaryl-Coenzyme A synthase 1 (soluble)	HMGCS1	siRNA protein
Q03426	mevalonate kinase	MVK	None
Q14534	squalene epoxidase	SQLE	Known drug target
P48449	lanosterol synthase (2 3-oxidosqualene-lanosterol cyclase)	LSS	Known drug target
Q15126	phosphomevalonate kinase	PMVK	None
P53602	mevalonate (diphospho) decarboxylase	MVD	None
O95749	geranylgeranyl diphosphate synthase 1	GGPS1	None
P37268	farnesyl-diphosphate farnesyltransferase 1	FDFT1	HIV-1 target according to predictions

Cholesterol efflux and cholesterol synthesis are different pathways, having in common that they link cholesterol to HIV-1 pathogenesis. Thus, our pathway analysis suggests that HIV-1 not only takes over control of cholesterol concentrations inside cells, but also cholesterol synthesis. This would not be surprising, since cholesterol is needed for HIV-1 virion formation, docking and fusion. Thus, one novel anti-HIV-1 drug treatment strategy suggested by our result would be to inhibit cholesterol synthesis, and drugs to test this hypothesis already exist (since the enzymes encoded by the LSS and SQLE genes are known drug targets).

## Conclusions and future work

We created a mapping of HIV-1, human interactions to human signal transduction pathways to link interactions with functions. We proposed a new way of analyzing virus-host interactions by identifying HIV-1 interaction partners in pathways as well as alternative paths bypassing the HIV-1 targeted steps in human signal transduction pathways. Due to spatial and temporal constraints, the fact that there is a human host factor in a pathway does not necessarily imply that this pathway is altered during infection. In particular, the strength and lifetime of an interaction between a human and an HIV-1 protein will determine what pathways may really have an effect that is lasting and transforming. Many proteins targeted by HIV-1 are hub proteins with a high degree of interactions. These interactions will not occur at the same time and in the same complex composition, so the effect that the interaction with the HIV-1 protein would have will vary over time and with regulation of each respective complex by posttranslational modifications, protein activity and localization. Thus, taking the view that interaction with an HIV-1 protein means that the signal transduction pathway is targeted is clearly simplistic. However, despite the use of these simplifying assumptions, the observation that a pathway contains a putative HIV-1 binding partner at least provides us with a testable hypothesis that HIV-1 interferes with normal cellular functioning via the respective pathway.

Future work may include taking the hierarchy of nodes in pathways into account in the analysis. This would allow us to study in detail where in the logical progression of a pathway the HIV-1 targets are located so as to gage to what extent putative drug targets are located up-or down-stream of these targets. This may influence how pharmacologically effective a treatment would be. For example, treatment strategies based on downstream targets may have fewer side-effects than up-stream targets. On the other hand, up-stream targets may be more amenable to pharmacological intervention, especially if they include cell surface receptors. Other definitions of input and output nodes can also be evaluated, such as linking them to molecular functions such as receptors (input) and transcription factors (output). Another approach could be to view the known drug targets as putative input nodes. While these are many viable future directions, using our current definition, the approach already yields numerous experimentally testable hypotheses on how HIV-1 function may be compromised and human cellular function restored by pharmacological approaches. We described some pathways in this paper, such as the cholesterol biosynthesis pathway, and our analysis provides proof-of-concept that we can find druggable pathways that may hold promise for anti-HIV drug discovery. There are many other pathways that may hold equal or more promise for drug discovery and we make available as a resource to the community the full set of HIV-1 path and alternative path statistics on pathways, as well as the lists of the points of HIV-1 interception of pathways. This resource will allow researchers to investigate these pathways in detail and derive complementary approaches to understand the mechanisms of and circumvent HIV-1 takeover of the cell.

## Methods

### Pathway data

We have collected hundreds of pathways from the National Cancer Institute (NCI) and Reactome protein pathway databases [12,15]. At the time of down-loading (November 2008), the NCI Pathway Interaction Database (PID) contained 83 hand-curated pathways containing nearly 5000 interactions. The Reactome database contained 823 hand-curated pathways and reported over 6000 interactions. The two databases display significant overlap and some pathways were merely textual descriptions of certain processes with insufficient detail into the actual members (proteins and small molecules) involved in the process. For our analysis, we therefore removed pathways which were complete subsets of other pathways and also removed pathways which did not contain sufficient detail for our experiments. The final dataset contained 453 pathways. For some of the pathways, a relationship to HIV biology is clear from the name, e.g. "HIV Nef pathway", "Tat-mediated elongation of the HIV-1 transcript", "Vif-mediated degradation of APOBEC3G", and HIV-1 proteins are listed as part of the pathways. We did not treat these pathways differently from any other pathway in the database only containing human proteins.

### Interactions data

To determine the intersection between the HIV-1, human interactome and the gathered signal transduction pathways, we need to define the interactome. We used the following 5 definitions: Group 1 are the most likely direct, physical known interactions based on reported literature [3], while Group 2 refer to more likely indirect interactions reported in the literature [3]. (For more details on definition of Group 1 and Group 2, see [9]). In a separate effort to define a better defined set of known interactions, we asked expert virologists to annotate the interactions from the literature. We found that many HIV-1 virologists do not trust the majority of the interactions reported in the literature. Expert validated targets 1 refers to those interactions where at least one expert annotated the interaction as real. Expert validated targets 2 refers to those interactions where at least two experts agree on their label. Finally, we included all of our predicted interactions [9].

### Path identification

For each pathway, we define simple paths that start with a protein that does not receive an input from another protein and end with a protein that does not induce a change in any other protein. Searching for simple paths in signal transduction pathways was accomplished as follows. Simple paths are those from a start node to an end node which have no repeating vertices, where a start node is defined as a node which has all its edges directed away from it and an end node is defined as one which has all its edges directed into it. The following example illustrates the way paths are counted. Assume you have the pathway consisting of interactions X → Y → Z, X → A → Z, B → A → Z, C → D → E, where Y is the HIV-1 targeted protein. According to our definition X, B, C are valid input nodes and Z, E are valid output nodes. Four is the total number of paths in this pathway. Then, the paths containing the HIV-1 targeted proteins are removed. Counting again, we would have 3 alternative paths left (namely X → A → Z, B → A → Z, C → D → E). This number answers the question how many of the complete paths (from a start node of the original graph to an end node of the original graph) are still "active" after HIV-1 infection. These paths are referred to as alternative paths throughout the text.

To find these simple paths in the full network graph of each signal transduction pathway we use a breadth first search algorithm [16], where we begin at the start nodes and produce their children recursively until we reach the end nodes, to find all the simple paths in the pathway graph. One immediate problem with a breadth first search is that it can get stuck in cycles (if a set of interactions forms a directed cycle then a simple breadth first search will not terminate). To handle this we maintain a list of nodes that have been visited in the particular simple path we are exploring and terminate if we re-visit a node. This ensures that for example a simple path with a simple cycle (a simple cycle is a cycle which has the same start and end vertex but no other vertex in the cycle repeats) will be explored as a single simple path which ignores the cycle. We further proceed to find paths that are not targeted by HIV-1 according to each of our datasets (we look at all the paths from above and ignore those that contain targeted proteins). We then identify which of these paths contain proteins that interact with HIV-1 proteins, and then find alternative paths between the same start and end points that do not traverse any protein that can interact with an HIV-1 protein.

### Drug target, siRNA and tissue expression data

We supplement the combined map of signal transduction pathways with HIV-1, human protein interactions with functional information, namely which proteins are known drug targets [17,18] and which proteins have shown an effect on HIV-1 infectivity and other functions upon siRNA silencing [5-7]. The drug targets were downloaded from DrugBank on May 1, 2009 [17,18]. All drug targets provided in the database were used, which comprised 5463 proteins. The source of the siRNA data was the three genome scale screens [5-7] and the data were downloaded from the supplementary materials. In each case, the final list of siRNA genes reported by the authors was used. To identify whether a gene is expressed in HIV-1 susceptible tissues or not, we retrieved the tissues in which human proteins are expressed from the Human Protein Reference Database (HPRD) [19] and the Human Proteinpedia (HUPA) website [20]. A total of 13920 human genes are annotated with at least one tissue according to HPRD and HUPA. The tissues susceptible to HIV-1 infection were obtained from Levy et al. [21].

## List of abbreviations used

AIDS: Acquired immune deficiency syndrome; HIV-1: human immunodeficiency virus type 1; EGFR: epidermal growth factor receptor. NIAID: National Institutes of Autoimmune and Infectious Diseases.

## Competing interests

The authors declare that they have no competing interests.

## Authors' contributions

Sivaraman Balakrishnan collected the pathway and drug target data and conducted the study. Oznur Tastan collected and predicted HIV-1, human protein interaction data along with siRNA data and participated in the analysis of the results presented here. Jaime Carbonell supervised computational aspects of this work. Judith Klein-Seetharaman proposed the alternative path idea, designed this study, analyzed the data collected and wrote the paper. All authors participated in the editing of this paper.

## Note

Other papers from the meeting have been published as part of *BMC Bioinformatics *Volume 10 Supplement 15, 2009: Eighth International Conference on Bioinformatics (InCoB2009): Bioinformatics, available online at http://www.biomedcentral.com/1471-2105/10?issue=S15.

## Supplementary Material

Additional file 1 Table S1. This file lists the human proteins in each pathway, along with the information if and if yes which HIV-1 proteins interact with them, if they are siRNA genes or if they are drug targets. Click here for file

Additional file 2 Table S2. This file contains statistics on number of paths, HIV-1, siRNA genes, and drug targets for pathways from the NCI PID and the Reactome databases. Click here for file
